# The Diet of Schoolboys

**Published:** 1896-07-25

**Authors:** 


### The Diet of Schoolboys.

### I.—INTRODUCTORY.

On few subjects has more ink been wasted than on the best foods for the healthy, and the evolution of the chemistry of digestion had not till recently advanced sufficiently to afford such a scientific foundation as we might desire. In the last ten years, however, Ewald, Boas, Einhorn, and others have thrown much light on the gastric stage of digestion, and the investigation of diabetes and alcoholism has resulted in the gain of a few principles of alimentation. It is now possible to lay down a scientific dietary for certain diseases, so as to meet a single difficulty of digestion or absorption; but the rules of diet best for the healthy are still largely based on unsystematic observations. There is an initial difficulty in the extraordinary diversity of diet used with success by people inhabiting different countries. Pure vegetarian races are found side by side with pure flesh-feeders in the same climates. A radical change in diet for a given race is brought about by political or economic conditions. Such has been the introduction of potatoes, sugar, and tea among the English, and the abolition of alcohol among Moslem nations. The proper time and interval for meals has been laid down with much gravity and the assumption of scientific knowledge, yet we find perhaps a third of the human race flourishing on two meals daily, others taking regularly three to five, and, it may be from habit, unable to work if their food is given otherwise. Some writers have gone so far as to argue that the actual diet of a given race has been evolved as the most suitable for it in its special environment, but this is manifestly untrue if we regard the changes brought about by fashion and economic conditions. It is clear, then, that the human body has enormous powers of adaptation, and can obtain sufficient nourishment from the most diverse food-stuffs, though it is influenced, no doubt, by ancestral habits and psychical causes. Though it is true that we can obtain the requisite number of grains of nitrogen and carbon from numerous easily-digested substances, we are closely tied down in recommending a dietary by the habits and fashions to which our patients are accustomed; otherwise disgust and nausea will neutralise our efforts. We have, then, in choosing a diet for schoolboys, to make the best we can of the common national dietary and modes of feeding, and bring them as near as possible to a scientific ideal suited to the end in view. That end is not that of the cattle-feeder, to obtain the most rapid growth and fattening, but primarily to obtain the most healthy and enduring physique of which the race is capable. The schoolboy has to carry on mental labour and considerable athletic toil, as well as to maintain his growth and development.

Many invaluable practical rules are given by Dr. Clement Dukes in his "Essentials of School Diet," founded on his observations of Rugby boys. Yet the difficulty of the problems involved is shown by the very tables of growth which he quotes as showing the effect of deficiency of food in preventing growth. Thus, over 7,000 individuals of the poorer classes are compared with an equal number of the better-fed as to their rate of growth in height and weight from 10 years of age; and the surprising fact comes out that, though the better-fed weigh some 22 lbs. more than the worse-fed labouring classes at 20 years of age, their excess in stature is only that which they possessed at ten years old, viz., three inches, which is very possibly hereditary. Thus good feeding seems to have little or no effect on stature, so far as these tables go.

It seems clear from the experience of Dr. Dukes and others, as well as from the results on animals, and from the analogy of Nature's food for the young—milk—that some excess of nitrogenous food should be supplied to growing individuals, and Dukes recommends for the boy of fifteen some 8 ounces of cooked meat daily, besides milk, eggs, cheese, &c., or a total of 12 ounces; but Savory, from his observations on 500 boys at Haileybury, finds that an individual on the average will rarely eat more than 4½ ounces of meat at dinner, and all authorities are opposed to meat suppers. Hence, if the 8 ounces of cooked, boneless meat are to be got in, some 3 ounces must be given at breakfast, but the amount is probably more than is needed. The Rugby boy gets in addition about 28 ounces of milk daily, besides smaller quantities of soup, fish, and cheese during the week. The carbohydrates are usually obtained in the form of bread of various kinds, of which Savory finds that more than 14 ounces daily are rarely eaten; potatoes to the amount of 8 ounces, and sugar, which is recognised as a most useful food, in spite of the disfavour with which it was formerly regarded. The fats so much disliked by children generally must be given as butter, or in suet puddings or as milk, and as the fat of meat to the amount of some 3 ounces daily. Mineral matter and vegetable salts must be supplied as green vegetables, fruits, and, if possible, by the use of wholemeal bread, though the difficulty of obtaining the latter in a form palatable to persons accustomed to white flour is considerable. In the Haileybury diet, which corresponds pretty closely to Parkes' dietary tables for individuals weighing 110 lbs., we find that the daily average amounts to 3·8 ounces of albuminates, 2·7 ounces of fats, 12 ounces of carbohydrates, and half an ounce of salts. The salts and fats here seem somewhat deficient, probably from difficulties in English cooking and the taste of boys. The amounts, of course, are reckoned without water, and correspond to about twice as much food in the state it is usually served.

### THE HERMITE SYSTEM AT NETLEY.

The Hermite system of sewage treatment, which was demonstrated at Worthing about two years ago and has been extensively practised on the Continent, has been adopted at the Netley Military Hospital. Important improvements have been introduced by the inventors for the first time. The electrolysed sea water is stored in a large tank at the top of the building, from whence pipes connect it with the whole system of drainage. The system is said to answer most admirably as a deodorising agent, and in preventing the accumulation of sewage slime in pipes and sewers. The work has been carried out by Messrs. Paterson and Cooper, Mr. Hermite's co-patentees in the process.

